# Heterologous Expression of Bacterial Dehydrin Promotes Arabidopsis Tolerance to Cadmium and Arsenic Stress

**DOI:** 10.3390/genes16121413

**Published:** 2025-11-27

**Authors:** Asmat Ali, Muhammad Usman, Waqar Ali, Nadir Zaman Khan, Muhammad Aasim, Nikola Staykov, Akhtar Ali, Iqbal Munir, Tsanko Gechev

**Affiliations:** 1 Department of Biotechnology, University of Malakand, Chakdara 18800, Pakistan; asmatams333@gmail.com (A.A.); usmangulab043@gmail.com (M.U.); nadir.zaman@uom.edu.pk (N.Z.K.); draasim@uom.edu.pk (M.A.); 2 Hefei University of Technology, Hefei 230009, China; 3 Institute of Biotechnology and Genetic Engineering, The University of Agriculture, Peshawar 25130, Pakistan; waqar.ali@aup.edu.pk; 4 Department of Molecular Stress Physiology, Center of Plant Systems Biology and Biotechnology, 4023 Plovdiv, Bulgaria; staykov@cpsbb.eu (N.S.); gultkr@yahoo.com (A.A.); 5 School of Advanced Biotechnology, Konkuk University, Seoul 05029, Republic of Korea; 6 Department of Molecular Biology, Plovdiv University, 4000 Plovdiv, Bulgaria

**Keywords:** cadmium stress, arsenic stress, dehydrin, proline, flavonoid, DPPH

## Abstract

Background: Abiotic stresses, such as drought, salinity, temperature fluctuations, waterlogging, and heavy metal contamination, have a detrimental impact on plants, leading to reduced global agricultural productivity. The accumulation of cadmium (Cd) and arsenic (As) in agricultural soil, resulting from both natural and anthropogenic activities, poses significant threats to crop production and food safety. Dehydrins, also known as Group II Late Embryogenesis Abundant (LEA) proteins, are intrinsically disordered proteins that play crucial roles in protecting cellular structures during abiotic stress conditions. These proteins are considered promising candidates for enhancing plant tolerance to environmental stresses through their membrane-stabilizing and protective functions. Methods: This study evaluated the tolerance of Arabidopsis transgenic lines expressing a bacterial dehydrin gene (*BG757*) to Cd and As stresses using various physiological and biochemical parameters. Results: Compared with the wild-type (WT) control, the transgenic line (35S::BG757-1/Col-0) displayed significant increases in root and shoot growth upon exposure to Cd and As. Furthermore, transgenic plants exposed to heavy metal stress exhibited higher concentrations of chlorophyll, total protein, free proline, total flavonoid, and total phenolic content compared to WT plants. Likewise, transgenic plants showed higher 2,2-diphenyl-1-picrylhydrazyl (DPPH) radical scavenging activity and retained higher relative water content under stress conditions. Conclusions: Taken together, these findings suggest that bacterial dehydrins confer enhanced tolerance to heavy metal stress in transgenic Arabidopsis plants, highlighting their potential application in developing stress-resilient crops for contaminated environments.

## 1. Introduction

Plants endure multiple environmental challenges that significantly constrain agricultural productivity worldwide. Among these, abiotic stresses, including extreme temperatures, salinity, drought, and heavy metal toxicity, severely impair crop growth and development by disrupting vital physiological processes such as ion homeostasis, macromolecular stability, and metabolic efficiency [1]. Heavy metal contamination represents a particularly critical abiotic stress factor, causing substantial yield losses while simultaneously posing serious health risks to human populations through bioaccumulation in the food chain [2]. Heavy metals such as cadmium (Cd), arsenic (As), lead (Pb), mercury (Hg), and chromium (Cr) are especially detrimental to plants, interfering with numerous metabolic pathways upon uptake [3]. These toxic elements originate from both natural and anthropogenic sources, including volcanic emissions, agrochemical applications (pesticides and fertilizers), industrial metal processing, vehicular emissions, residential effluents, and atmospheric deposition [4]. At the cellular level, heavy metals primarily disrupt interorgan communication and cell-to-cell signaling networks by directly targeting key signaling components including respiratory burst oxidase homologs (RBOHs), hormone biosynthesis and transport machinery, calcium (Ca^2+^) sensors, and mitogen-activated protein kinase (MAPK) cascades [5,6,7]. These primary disruptions to signaling pathways compromise organ-to-organ coordination and intercellular communication, leading to secondary consequences including oxidative damage, competitive displacement of essential cofactors, inhibition of enzymatic activities, compromised membrane integrity, altered gene transcription, and disruption of electron transport chains in chloroplasts and mitochondria as downstream effects [6,8]. Among the spectrum of heavy metal pollutants, cadmium and arsenic have been identified as the most prevalent and hazardous non-essential elements affecting both plants and mammals [9,10].

Cadmium exerts its phytotoxicity through multiple mechanisms. Cadmium directly activates RBOH-mediated reactive oxygen species (ROS) production as a primary signaling event, which then triggers downstream oxidative stress cascades [11]. Cadmium accumulation inhibits Fe (III) reductase activity, creating iron deficiency that directly impairs photosynthetic efficiency. Furthermore, Cd interferes with the uptake and translocation of essential nutrients including calcium, phosphorus, potassium, magnesium, and water, while simultaneously suppressing nitrate assimilation by inhibiting nitrate reductase [4]. At the molecular level, Cd binds to sulfhydryl groups on structural proteins, causing protein misfolding and disrupting redox-dependent processes, particularly those involving electron transport chains [12].

Arsenic exists in two predominant oxidation states: arsenite [As (III)] and arsenate [As (V)]. Arsenite is typically found under reducing conditions in subsurface soils and groundwater, whereas arsenate predominates in oxidized soil and water systems [13]. This toxic metalloid severely restricts plant growth and productivity, as plants readily absorb arsenic from contaminated soil and water, subsequently accumulating it in their tissues [14].

In response to abiotic stresses, plants have evolved sophisticated molecular defense mechanisms, including the expression of late embryogenesis abundant (LEA) proteins. These stress-responsive genes are widely distributed across plant species and exhibit upregulated expression during seed maturation and under stress conditions [15]. LEA proteins are classified into six groups based on sequence homology, with Group 2 “dehydrins” being among the most extensively characterized [16,17]. Dehydrins are highly hydrophilic, intrinsically disordered proteins induced by cellular dehydration resulting from various stresses including drought, freezing, salinity, osmotic stress, and hormonal signals such as abscisic acid and jasmonic acid [18]. During seed development, dehydrins and LEA proteins exhibit cell-type-specific expression patterns, with high accumulation in embryo tissues during late maturation stages, lower and compositionally distinct expression in endosperm, and specific localization to maternal nucellus tissue in some species [19,20]. Emerging evidence suggests that dehydrins possess metal-binding capabilities, potentially functioning as metal chelators to mitigate heavy metal toxicity in plants [21]. Under water-deficit conditions, changes in intracellular hydration status alter the spatial organization of macromolecular complexes, potentially leading to protein aggregation, aberrant molecular interactions, and denaturation of proteins and membrane structures [22,23]. Dehydrins counteract these detrimental effects through their amphipathic α-helical domains, which enable them to interact with other proteins and biomembranes, thereby functioning as molecular shields or chaperones under dehydration stress [24]. Several studies have demonstrated enhanced heavy metal tolerance in plants overexpressing dehydrin genes. For instance, overexpression of *BjDHN2* and *BjDHN3* in *Brassica juncea* by the constitutive CaMV35S promoter resulted in increased Cd^2+^ and Zn^2+^ accumulation in roots under metal stress, indicating improved metal tolerance [18]. On the other hand, antisense *BjDHN3 B. juncea* plants showed higher sensitivity to the two heavy metals [18]. Similarly, exposure of bean plants to mercury and cadmium induced elevated mRNA levels of the SK_n_-type dehydrin *PvSR3* [25], while aluminum stress upregulated *Dhn4* expression in barley roots [26].

Beyond plants, microorganisms have demonstrated remarkable stress tolerance capabilities. Recent research has shown that heterologous expression of a putative bacterial dehydrin in *Arabidopsis thaliana* enhanced drought and salinity tolerance [27], highlighting the potential of microbial stress-responsive genes for crop improvement. Building upon this foundation, the present study investigates whether heterologous expression of the bacterial dehydrin gene *BG757* can confer tolerance to cadmium and arsenic stress in plants, thereby expanding the functional repertoire of microbial dehydrins in agricultural biotechnology.

## 2. Materials and Methods

### 2.1. Plant Material and Growth Conditions

The transgenic line (BG757-1) and WT (Columbia/Col-0 wild type Arabidopsis) seeds of *A. thaliana* were provided by the Plant Biotechnology Lab, Department of Biotechnology, University of Malakand, Chakdara, Pakistan. The selected transgenic line (BG757-1) was used from our previously reported study [23].

Half-strength Murashige and Skoog (½ MS) medium was prepared by dissolving 2.2 g L^−1^ MS basal salt mixture, 5 g L^−1^ sucrose, and 0.5 g L^−1^ MES hydrate buffer in distilled water (total volume 1 L). The pH was adjusted to 5.8 using 1 M KOH, and 8 g L^−1^ (1%) agarose was added for solidification. The medium was autoclaved at 121 °C for 20 min at 15 psi. After autoclaving, the molten medium was poured into 100 mL-capacity flasks and allowed to solidify. For stress treatments, the media were supplemented with 50 and 80 µM cadmium (Cd) or 10 and 15 µM arsenic (As), following previously reported concentrations [28,29]. Control media were prepared under identical conditions but without Cd or As supplementation

The seeds of *A. thaliana*, both WT and the transgenic line, were surface sterilized [30] and sown on half-strength MS media. The seeds were sterilized using two solutions: Solution 1, consisting of 0.5% (*v*/*v*) Triton X-100 and 70% (*v*/*v*) ethanol, was applied for five minutes, followed by Solution 2, containing 100% ethanol, for ten minutes. The sterilized seeds were then sown on the solidified half-strength MS media contained in the 100 mL flasks. The flasks were placed in a growth chamber and maintained under controlled conditions of 25 ± 1 °C temperature, 75 ± 5% relative humidity, a photosynthetic photon flux density (PPFD) of 120 μmol m^−2^ s^−1^ provided by cool-white fluorescent lamps, and a 16 h light/8 h dark photoperiod for three weeks.

### 2.2. Assessment of Root and Shoot Length

Plants were harvested three weeks post-stress treatment. Root and shoot lengths were measured in triplicate for both control and stress-exposed plants (Cd: 50 µM and 80 µM; As: 10 µM and 15 µM). Data are presented as mean ± standard deviation of three biological replicates with three technical replicates each. Statistical analysis was performed using one-way ANOVA followed by Tukey’s post hoc test in GraphPad Prism 8.01 software.

### 2.3. Fresh Weight and Dry Weight

Fresh tissues were weighed immediately upon collection using a physical balance. Dry weight was determined after drying tissues at 60 °C for 48 h in a microwave oven [31].

### 2.4. Relative Water Content Determination

Relative water content (RWC) was measured using fresh leaves for both transgenic and WT plants, utilizing the previously reported method [32]. By utilizing the following formula, the RWC was determined.

RWC = (fresh weight − dry weight)/(turgor weight − dry weight) × 100

### 2.5. Assessment of Secondary Metabolism and Radical Scavenging Activity

#### 2.5.1. Secondary Metabolite Extraction

Dried plant tissue (300 mg) was collected in a 1.5 mL Eppendorf tube and mixed vigorously with 1.5 mL absolute methanol. The mixture was incubated for 5 min, followed by sonication for 5 min and vortexing. Samples were then centrifuged at 13,000 rpm for 5–7 min. The supernatant was transferred to fresh tubes and left open to allow methanol evaporation, resulting in dry residue accumulation [33].

#### 2.5.2. Quantification of Total Phenolic Content

For TPC analysis, Folin–Ciocalteu (FC) (BDH Laboratory Supplies, Poole, Dorset, UK; Prod. No. 19058 2P) reagent, sodium carbonate (Na_2_CO_3_), and gallic acid (Sigma-Aldrich, St. Louis, MO, USA; CAS No. 149-91-7) were used. Stock solutions were prepared as follows: FC reagent was dissolved in deionized water, sodium carbonate solution was prepared at 6% *w*/*v* in distilled water (6 g/100 mL), and gallic acid was dissolved in methanol at a concentration of 4 mg/mL. Using a multi-channel micropipette, 20 µL of sample and 90 µL of FC reagent were added to a 96-well plate and incubated for 5 min. Subsequently, 90 µL of Na_2_CO_3_ was added to each well. Gallic acid solutions at varying concentrations (5, 10, 15, 20, 25 µg/mL) were used as positive controls. Absorbance readings were obtained at 630 nm using a microplate reader (TSx800 Absorbance Reader, BioTek Inc., Shoreline, WA, USA).

Total flavonoid content was quantified using aluminum chloride, quercetin, and potassium acetate. Stock solutions were prepared by dissolving quercetin at 4% *w*/*v* in methanol, potassium acetate at 1 M in distilled water, and aluminum chloride at 10% *w*/*v*. Using a multi-channel micropipette, 20 µL of sample, 10 µL of aluminum chloride, 10 µL of potassium acetate, and 160 µL of distilled water were added to 96-well plates and incubated for 30 min. Two controls were included: a positive control with quercetin at concentrations of 2.5, 5, 10, 20, and 40 µg/mL, and a negative control with 20 µL of methanol used in place of the sample. Absorbance readings were collected at 405 nm using a microplate reader (TSx800 Absorbance Reader, BioTek Inc., Shoreline, WA, USA) [33].

#### 2.5.3. Determination of Radical Scavenging Assay

The DPPH (diphenyl picryl hydrazyl) radical scavenging assay was performed using DPPH reagent and ascorbic acid. Stock solutions were prepared by dissolving DPPH reagent (3.2 mg) and ascorbic acid (4 µg/mL) in 100 mL of methanol. Using a multi-channel micropipette, 20 µL of sample and 180 µL of DPPH reagent were added to 96-well microplates. The plates were then incubated for 60 min in the dark. Absorbance readings were measured at 517 nm using a microplate reader (TSx800 Absorbance Reader, BioTek Inc., USA) [34].

DPPH scavenging activity (%)/% Inhibition = A0 − A1/A0 × 100

Where A0 = absorbance of control

A1 = absorbance of standard.

### 2.6. Assessment of Free Proline Content

To assess free proline content, 100 mg of freshly harvested leaves were rinsed with deionized water, homogenized using a mortar and pestle, and transferred to a 2 mL Eppendorf tube containing 3% sulfosalicylic acid. After thorough mixing, the samples were centrifuged for 5 min at 13,000 rpm. The supernatant was transferred to a 15 mL falcon tube, and ninhydrin reagent along with 6 M orthophosphoric acid was added. The tubes were incubated in a water bath at 100 °C for 1 h. Following incubation, the samples were immediately placed in ice-cold water to terminate the reaction. Subsequently, 4 mL of toluene was added to each sample and mixed vigorously using a separating funnel. After adequate shaking, two distinct phases appeared in the funnel. The upper pink layer was collected, and its optical density was measured at 520 nm using a spectrophotometer. Toluene was used as the blank control [35].

### 2.7. Quantification of Photosynthetic Pigments

For photosynthetic pigment quantification, approximately 100 mg of fresh leaves were collected for each sample, rinsed with deionized water, and homogenized using a mortar and pestle. The ground tissue was transferred to 2 mL Eppendorf tubes and mixed with 80% acetone for 60 min. The samples were subsequently centrifuged at 13,000 rpm for 5 min at 4 °C. Following centrifugation, the supernatant was collected and diluted to a final volume of 5 mL with 80% acetone in 15 mL falcon tubes. Absorbance readings for chlorophyll a, chlorophyll b, and carotenoids were measured using a spectrophotometer at wavelengths of 663 nm, 645 nm, and 470 nm, respectively [36]. The following formulas were used to calculate the values.

Chlorophyll *_a_* (μg/mL) = 12.25 (A_663) − 2.55 (A_645)

Chlorophyll *_b_* (μg/mL) = 20.31 (A_645) − 4.91 (A_663)

Total chlorophyll *_total _* (μg/mL) = 17.76 (A_645) + 7.34 (A_663)

Carotenoids *_C_* (μg/mL) = (1000*A_470 − 3.27*chl a) − ((1.04*chl b)/227)

### 2.8. Total Protein Quantification

For total protein quantification, fresh leaves were homogenized using a mortar and pestle. Proteins were extracted using an extraction buffer containing Tris-HCl (50 mM, pH 6.8), glycerol (5% *v*/*v*), SDS (0.5% *w*/*v*), Triton X-100 (1% *v*/*v*), DTT (10 mM), and EDTA (5 mM), with protease inhibitor cocktail added at 1 mL per 100 mL of buffer. Following extraction, the samples were centrifuged at 20,000 rpm at 4 °C. The supernatant was collected, and protein concentration was determined by measuring the optical density at 595 nm using a spectrophotometer (Thermo Fisher Scientific, Madison, WI, USA). Bradford reagent was used as the standard for protein quantification [37].

### 2.9. Statistical Analysis

Average of three replicates together with standard deviation are used to present the data. The Tukey test and two-way ANOVA were employed to compare treatment means. Version 8.01 of Graph Pad Prism was used for analysis.

## 3. Results

### 3.1. Transgenic Plants Expressing Bacterial Dehydrin Gene BG757 Grow Better Under Cd and as Stress

**Figure 1:**
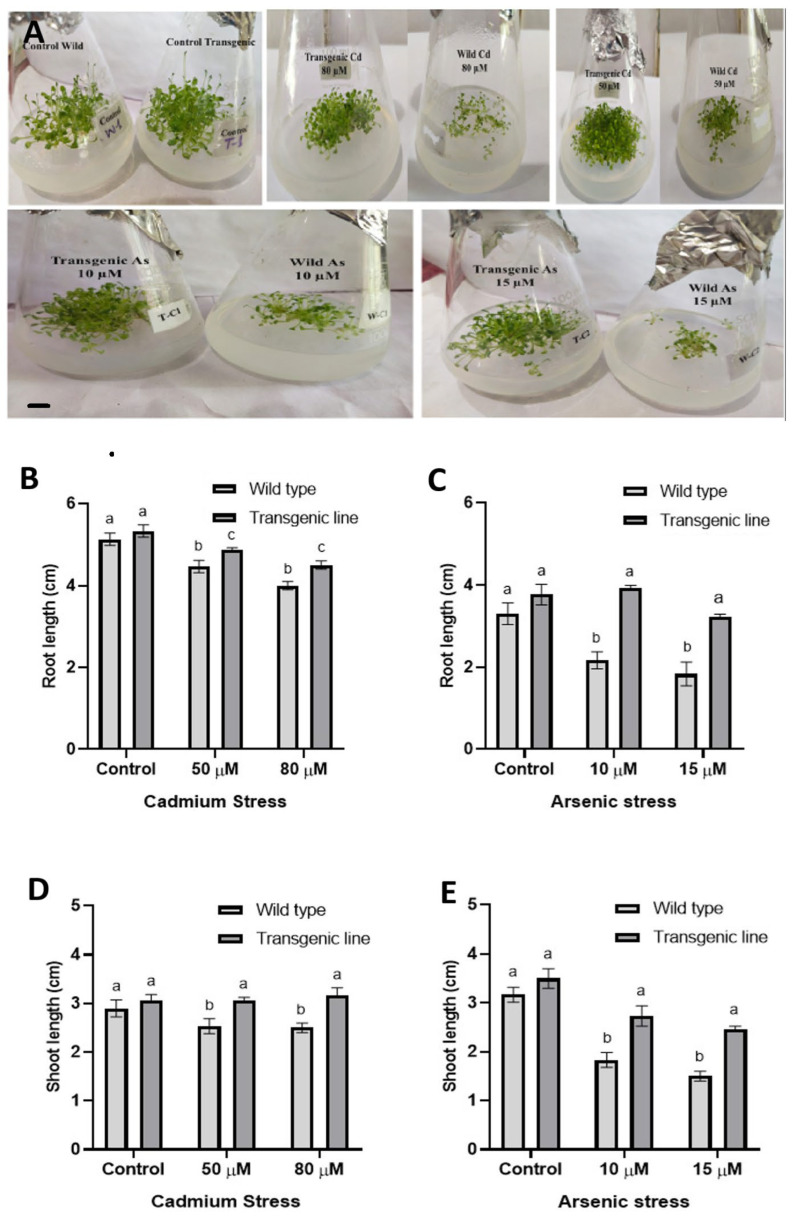
**Phenotype of wild-type (WT) and transgenic *A. thaliana* plants expressing BG757 under heavy metal stress.** (**A**) Representative pictures of WT and the transgenic line under normal conditions, Cd, and As stress. Pictures are taken three weeks after germination. The scale bar in the lower left corner indicates 1 cm. (**B**,**C**) Root length of WT and transgenic plants after three weeks of exposure to (**B**) Cd (50 and 80 μM) and (**C**) As (10 and 15 µM). (**D**,**E**) Shoot (rosette) length after three weeks of exposure to (**D**) Cd (50 and 80 μM) or (**E**) As (10 and 15 µM). Data represent means ± SEM (*n* = 3); different letters indicate significant differences (*p* < 0.05).

In prior studies, we demonstrated that the expression of the bacterial dehydrin gene (*BG757*) in *A. thaliana* enhances tolerance to drought and salinity [23]. Accordingly, we sought to investigate whether *BG757* also plays a role in conferring tolerance to heavy metals. Compared with WT, the *BG757* transgenic line grew much larger under cadmium (Cd) and arsenic (As) stress (Figure 1A).

Under Cd stress, transgenic plants exhibited significantly increased root length compared to WT plants (4% increase at 50 µM and 7% at 80 µM), while both genotypes showed comparable root lengths in control medium (Figure 1B). Similarly, after three weeks of As exposure, transgenic lines showed substantial root length enhancement (37% increase at 10 µM and 23% at 15 µM) relative to WT plants (Figure 1C). Subsequently, we examined the rosette leaves (shoot) responses of WT and transgenic plants under Cd and As stress. As expected, Cd and As exposure substantially inhibited rosette growth in WT plants. In contrast, transgenic plants maintained greater shoot lengths under both stress conditions: 13% increase at 50 µM Cd and 17% at 80 µM Cd (Figure 1D). Under As stress, transgenic plants showed 21% and 23% increases in shoot length at 10 µM and 15 µM, respectively, compared to WT plants (Figure 1E). Collectively, these results indicate that heterologous expression of bacterial *BG757* dehydrin gene enhances heavy metal tolerance in *A. thaliana*.

### 3.2. Transgenic Plants Retain Higher Relative Water Content in the Leaf Tissue Under Cd and as Stress

**Figure 2:**
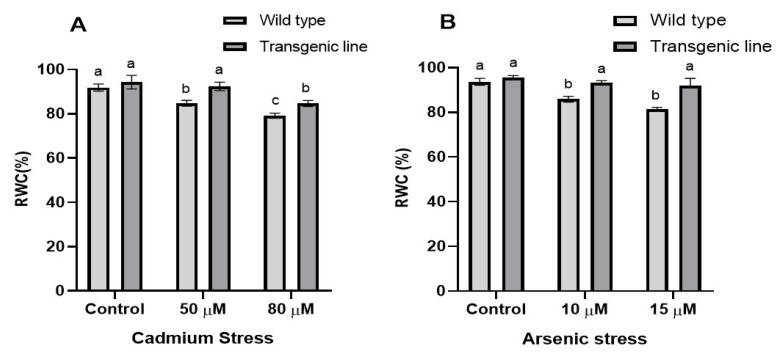
**Relative water content in WT and transgenic line under cadmium and arsenic stress.** Relative water content in the leaf tissues of transgenic *A. thaliana* with the dehydrin gene exposed to three weeks of (**A**) cadmium stress (50 μM and 80 μM) and (**B**) arsenic stress (10 µM and 15 µM). Data represent means ± SEM (*n* = 3); different letters indicate significant differences (*p* < 0.05).

We examined the relative water content (RWC) in WT and transgenic lines subjected to cadmium (Cd) and arsenic (As) stress. The relative water content in the leaf tissue of both the transgenic line and WT did not reveal any significant differences under control conditions. However, notable variations in RWC were detected under both Cd and As stress. According to the findings, the transgenic lines demonstrated significantly elevated relative water content (RWC) under cadmium stress, retaining more moisture (5% at 50 μM Cd and 3% at 80 μM Cd) compared to WT plants. A similar pattern was observed in plants exposed to arsenic, where the transgenic lines maintained significantly higher relative water content (RWC) than WT at both tested arsenic concentrations (6% at 10 μM and 3% at 15 μM) (Figure 2A,B).

### 3.3. Transgenic Plants Accumulate More Proline, Phenolic and Flavonoid Content Under Cd and as Stress

**Figure 3:**
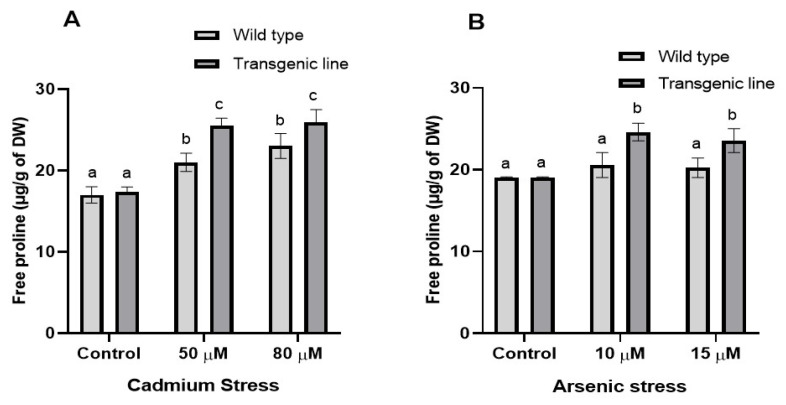
**Proline accumulation under cadmium and arsenic stress.** Free proline concentration in leaf tissues of transgenic *A. thaliana* with the dehydrin gene exposed to three weeks of (**A**) cadmium stress (50 μM and 80 μM) and (**B**) arsenic stress (10 µM and 15 µM). Data represent means ± SEM (*n* = 3); different letters indicate significant differences (*p* < 0.05).

**Figure 4:**
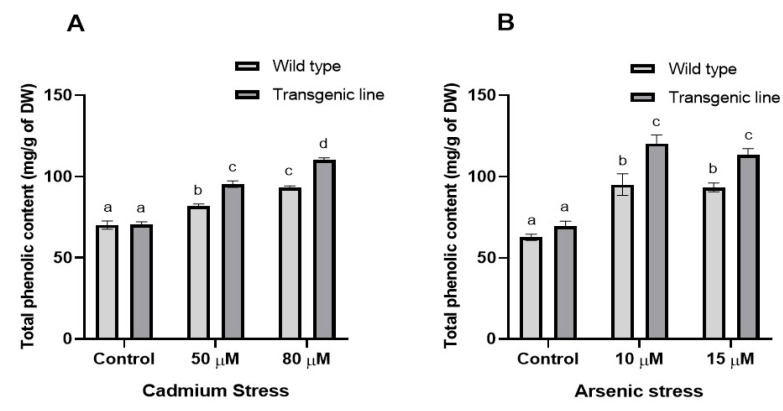
**Total phenolic content in WT and transgenic line under cadmium and arsenic stress.** Total phenolic content in leaf tissues of transgenic *A. thaliana* with the dehydrin gene exposed to three weeks of (**A**) cadmium stress (50 μM and 80 μM) and (**B**) arsenic stress (10 µM and 15 µM). Data represent means ± SEM (*n* = 3); different letters indicate significant differences (*p* < 0.05).

Both transgenic and WT plants showed increased proline accumulation after three weeks of Cd and As exposure, although proline levels were similar under control conditions. Under Cd stress, transgenic lines accumulated significantly more proline (23% increase at 50 µM and 14% at 80 µM) compared to WT plants (Figure 3A). Similarly, under As stress, transgenic lines showed 21% and 17% higher proline content at 10 µM and 15 µM, respectively (Figure 3B). Total phenolic content differed significantly between WT and transgenic lines under both stress conditions. Transgenic plants exhibited substantially higher phenolic content than WT plants, with increases of 16% and 24% at 50 µM and 80 µM Cd, respectively, and 22% and 14% increases at 10 µM and 15 µM As, respectively (Figure 4A,B).

**Figure 5:**
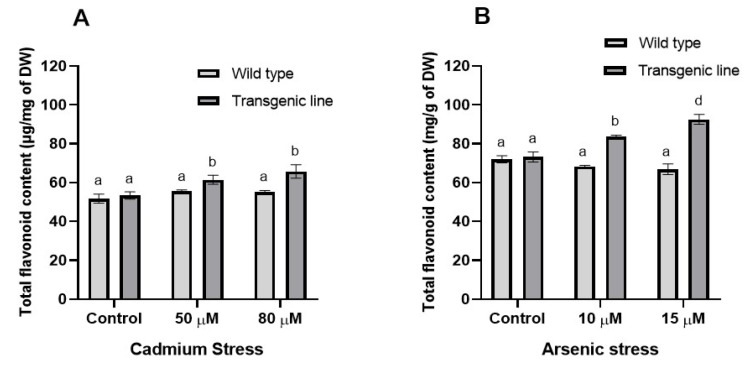
**Flavonoid content.** Total flavonoid content in leaf tissues of transgenic *A. thaliana* with the dehydrin gene exposed to three weeks of (**A**) cadmium stress (50 μM and 80 μM) and (**B**) arsenic stress (10 µM and 15 µM). Data represent means ± SEM (*n* = 3); different letters indicate significant differences (*p* < 0.05).

Total flavonoid content in leaf tissues was comparable between WT and transgenic plants under control conditions but differed markedly under stress. Transgenic plants showed significantly higher flavonoid content during Cd stress (7% and 17% increases at 50 µM and 80 µM, respectively). Under As stress, transgenic plants maintained 20% and 34% higher flavonoid content at 10 µM and 15 µM, respectively, compared to WT plants (Figure 5A,B).

### 3.4. DPPH Free Radical Scavenging Activity Increases in Transgenic Line upon Cd and as Stress

**Figure 6:**
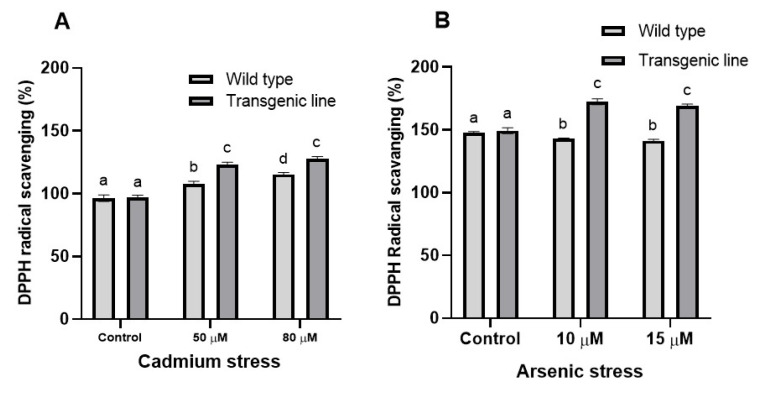
**DPPH free radical scavenging activity under cadmium and arsenic stress.** DPPH free radical scavenging activity in the leaf tissues of transgenic *A. thaliana* with the dehydrin gene exposed to three weeks of (**A**) cadmium stress (50 μM and 80 μM) and (**B**) arsenic stress (10 µM and 15 µM). Data represent means ± SEM (*n* = 3); different letters indicate significant differences (*p* < 0.05).

DPPH free radical scavenging activity was comparable between transgenic and WT plants under control conditions. However, after three weeks of Cd and As exposure, transgenic lines exhibited significantly enhanced scavenging capacity. Under Cd stress, transgenic plants showed 15% and 12% higher DPPH radical scavenging activity at 50 µM and 80 µM, respectively, compared to WT plants. Under As stress, transgenic plants demonstrated greater radical scavenging activity (19% and 18% increases at 10 µM and 15 µM, respectively) while WT plants showed declining activity (Figure 6A,B).

### 3.5. Photosynthetic Pigments and Carotenoid Content Decrease When Exposed to Cd and as Stress

**Figure 7:**
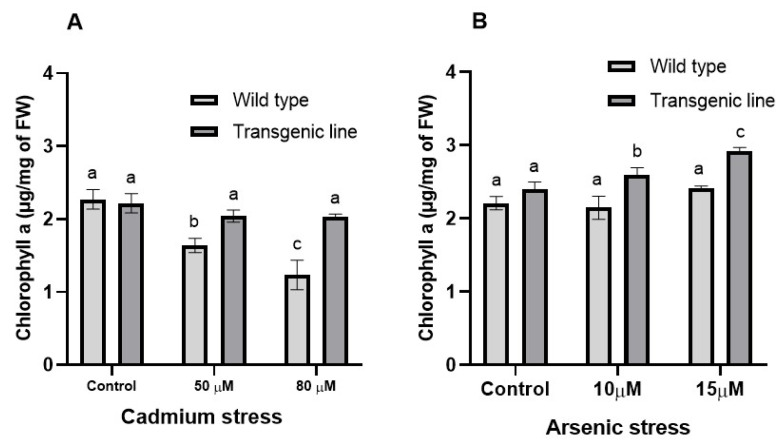
**Chlorophyll a in WT and transgenic plants under cadmium and arsenic stress.** Chlorophyll a in the leaf tissues of transgenic *A. thaliana* with the dehydrin gene exposed to three weeks of (**A**) cadmium stress (50 μM and 80 μM) and (**B**) arsenic stress (10 µM and 15 µM). Data represent means ± SEM (*n* = 3); different letters indicate significant differences (*p* < 0.05).

Chlorophyll ‘a’ content was comparable between transgenic and WT plants under control conditions. However, after three weeks of Cd exposure, WT plants experienced substantial chlorophyll ‘a’ reduction, while transgenic plants maintained significantly higher levels (20% and 37% increases at 50 μM and 80 μM Cd, respectively) compared to WT plants (Figure 7A). Similarly, under As stress, transgenic lines maintained 11% and 13% higher chlorophyll ‘a’ content at 10 μM and 15 μM, respectively (Figure 7B).

**Figure 8:**
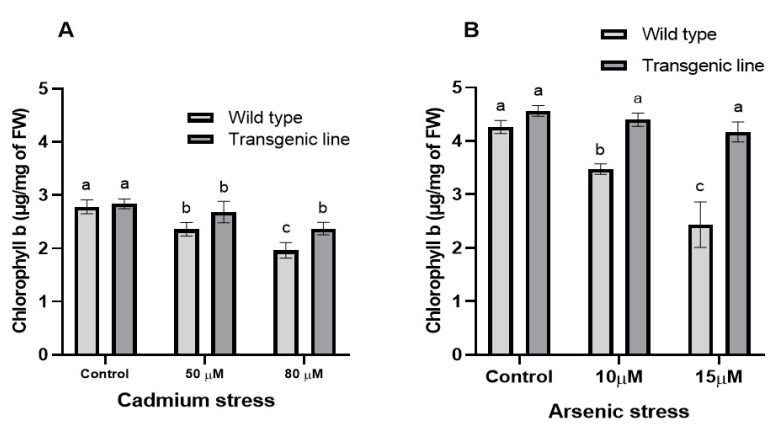
**Chlorophyll b in WT and transgenic plants under cadmium and arsenic stress.** Chlorophyll b in the leaf tissues of transgenic *A. thaliana* with the dehydrin gene exposed to three weeks of (**A**) cadmium stress (50 μM and 80 μM) and (**B**) arsenic stress (10 µM and 15 µM). Data represent means ± SEM (n = 3); different letters indicate significant differences (*p* < 0.05).

Chlorophyll ‘b’ content decreased significantly in both transgenic and WT plants under Cd or As exposure. Nevertheless, transgenic plants maintained markedly higher chlorophyll ‘b’ levels than WT plants: 10% and 15% increases at 50 μM and 80 μM Cd, respectively, and 15% and 34% increases at 10 μM and 15 μM As, respectively (Figure 8A,B).

**Figure 9:**
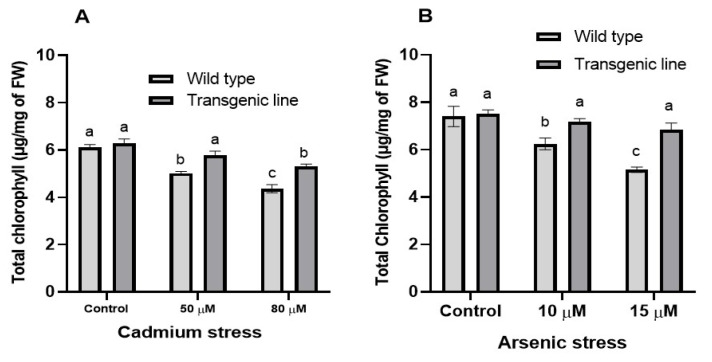
**Total chlorophyll content under cadmium and arsenic stress.** Total chlorophyll content in leaf tissues of transgenic *A. thaliana* with the dehydrin gene exposed to three weeks of (**A**) cadmium stress (50 μM and 80 μM) and (**B**) arsenic stress (10 µM and 15 µM). Data represent means ± SEM (*n* = 3); different letters indicate significant differences (*p* < 0.05).

Consistent with chlorophyll ‘a’ and ‘b’ results, total chlorophyll content decreased significantly in both genotypes under Cd or As stress. However, transgenic plants maintained substantially higher total chlorophyll content than WT plants: 10% and 13% increases at 50 μM and 80 μM Cd, respectively, and 11% and 22% increases at 10 μM and 15 μM As, respectively (Figure 9A,B).

**Figure 10:**
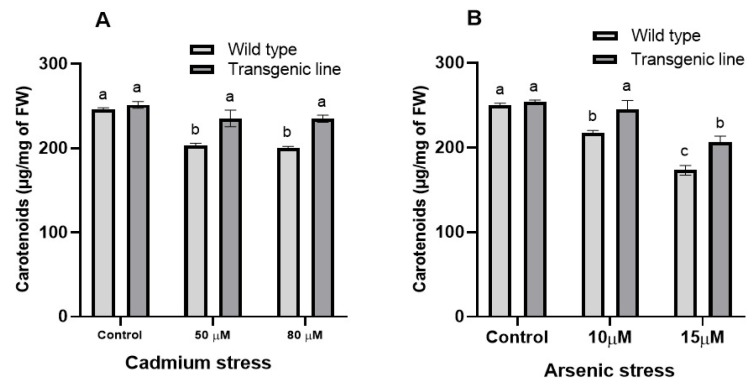
**Carotenoid content in under cadmium and arsenic stress.** Carotenoid content in leaf tissues of transgenic *A. thaliana* with the dehydrin gene exposed to three weeks of (**A**) cadmium stress at 50 μM and 80 μM and (**B**) arsenic exposure at 10 µM and 15 µM. Data represent means ± SEM (*n* = 3); different letters indicate significant differences (*p* < 0.05).

Carotenoid content declined with increasing Cd or As concentrations. Under control conditions, carotenoid levels were comparable between transgenic and WT plants. However, under Cd stress, transgenic plants maintained significantly higher carotenoid levels than WT plants (11% and 12% increases at 50 μM and 80 μM, respectively) (Figure 10A). Similarly, under As stress, transgenic plants preserved 10% and 13% higher carotenoid levels at 10 μM and 15 μM, respectively, despite comparable levels under control conditions (Figure 10B).

### 3.6. Effects on Total Protein Content Under Cd and as Stress

**Figure 11:**
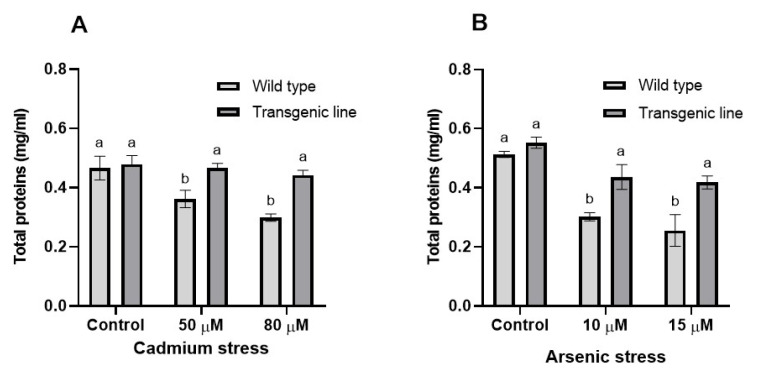
**Total protein content in WT and transgenic lines under cadmium and arsenic stress.** Total protein content in the leaf tissues of transgenic *A. thaliana* with the dehydrin gene exposed to three weeks of (**A**) cadmium stress (50 μM and 80 μM) and (**B**) arsenic stress (10 µM and 15 µM). Data represent means ± SEM (*n* = 3); different letters indicate significant differences (*p* < 0.05).

Transgenic Arabidopsis thaliana plants expressing BG757 showed substantially higher total protein content than WT plants following Cd exposure (20% and 28% increases at 50 μM and 80 μM Cd, respectively) (Figure 11A). No significant differences in total protein content were observed between transgenic and WT plants under control conditions. Under As stress, transgenic plants exhibited notably higher protein content than WT plants (21% and 26% increases at 10 μM and 15 μM As, respectively) (Figure 11B).

## 4. Discussion

Heavy metals like cadmium (Cd) and arsenic (As) accumulate in agricultural soils due to human activities and natural processes, posing serious risks to crop growth. These metals disrupt plant physiology, leading to stress and reduced productivity. To cope, plants produce stress-responsive proteins, including Late Embryogenesis Abundant (LEA) proteins, which enhance tolerance to abiotic stresses such as drought, salinity, and heavy metal toxicity. LEA proteins have been shown to improve stress resistance in plants like *A. thaliana*, *Brassica napus*, and rice by stabilizing cellular structures and maintaining homeostasis [23,38]. These proteins have been grouped into six classes based on sequence similarities. The most significant LEA protein is Group 2 LEA, sometimes referred to as dehydrin protein, which has been shown to rise as a result of exposure to different stresses [17]. Under unfavorable conditions, dehydrin is thought to function as a chaperone, stabilizing membranes, vesicles, and protein structures in plants [16].

The bacterial dehydrin gene *BG757-1* was identified through in silico analysis, cloned into *A. thaliana*, and confirmed by PCR amplification. Transgenic lines expressing *BG757* showed improved resistance to salinity and drought stress. Although these plants were more sensitive to NaCl and ABA during germination and root growth, they demonstrated significant drought tolerance and higher levels of stress-inducible genes (*DREB2A*, *RD22*, *RD26*, *LEA7*, and *SOS1*), suggesting that *BG757* positively regulates responses to salt stress and ABA, enhancing overall stress tolerance [27]. Building on this foundation, the present study investigated the role of *BG757* in conferring tolerance to Cd and As stress in transgenic *A. thaliana*, thereby expanding our understanding of cross-kingdom stress protein functionality.

The heterologous expression of stress-related genes has emerged as a powerful strategy for engineering abiotic stress tolerance in plants. Recent studies demonstrate that bacterial stress proteins can function effectively in plant systems: bacterial cold-shock proteins from Arthrobacter sp. conferred cold, drought, and salt tolerance when expressed in rice [39]. Conversely, plant dehydrins can enhance stress tolerance in bacterial systems, as demonstrated by the tobacco dehydrin *NtDHN17*, which improved copper and oxidative stress tolerance in Escherichia coli through anti-aggregation mechanisms [40]. These bidirectional functional transfers underscore the evolutionary conservation of stress protection mechanisms across kingdoms [41]. However, direct comparisons between bacterial-derived and plant-derived dehydrins in identical plant systems remain limited. The present study addresses this gap by demonstrating that bacterial dehydrin *BG757* confers heavy metal tolerance in *A. thaliana*, providing novel evidence for cross-kingdom applicability of bacterial stress proteins in plant heavy metal defense.

Transgenic *A. thaliana* lines expressing *BG757* exhibited a high level of growth under Cd and As stress, with root length increasing by up to 37% compared to wild-type (WT) plants. This enhancement surpasses that observed in transgenic lines expressing plant-derived dehydrins *HbDHN1* and *HbDHN2* under similar stress conditions [18]. The improved root and shoot growth in *BG757* lines suggests effective management of reactive oxygen species (ROS), which are primary mediators of heavy metal toxicity. Heavy metals induce excessive ROS production, leading to lipid peroxidation, protein oxidation, and DNA damage, ultimately inhibiting plant growth [42]. Recent evidence indicates that dehydrins mitigate ROS-induced damage through multiple mechanisms: they activate antioxidant defense systems (increasing ascorbate, glutathione, and antioxidant enzymes such as CAT, SOD, and POD) and protect cellular components from oxidative damage [43,44]. The overexpression of rice LEA protein *OsLEA1a* reduced lipid peroxidation and enhanced antioxidant enzyme activities under CuSO_4_ stress, demonstrating direct ROS detoxification capacity [44]. Similarly, the *ApSK3* dehydrin from *Agapanthus praecox* reduced H_2_O_2_ and hydroxyl radical accumulation while upregulating antioxidant enzymes [43]. Our results suggest that *BG757* likely employs analogous mechanisms to reduce ROS accumulation, thereby maintaining cellular integrity and promoting growth under heavy metal stress. On the other hand, ROS is important for auxin distribution in roots and necessary for root growth [45].

Transgenic lines expressing *BG757* maintained significantly higher relative water content (RWC) under Cd and As stress (up to 6% increase compared to WT), consistent with the established role of dehydrins in water molecule binding and membrane stabilization. The amphipathic α-helical structure of dehydrins enables interaction with membrane phospholipids, reducing membrane permeability and water loss [46]. Recent biophysical studies confirm that conserved K-segments in dehydrins mediate membrane association and lower membrane phase-transition temperatures, providing cryoprotection and reducing electrolyte leakage. Under heavy metal stress, membrane integrity is compromised by ROS-induced lipid peroxidation. Dehydrins counteract this damage through direct membrane interaction and by reducing oxidative modifications of membrane lipids [47]. Notably, some dehydrins chelate metal ions (Cu^2+^, Fe^3+^), further protecting membranes from metal-catalyzed oxidative damage (Huang et al., 2022), although other dehydrins confer protection without direct metal binding, suggesting multiple protective mechanisms [43]. The enhanced RWC observed in *BG757*-expressing plants likely reflects both membrane stabilization and reduced oxidative membrane damage, enabling better water retention under stress.

Transgenic lines expressing *BG757* maintained significantly higher levels of chlorophyll a, chlorophyll b, carotenoids, and total chlorophyll under Cd and As stress compared to WT. This protective effect exceeded that reported for plant-derived dehydrins: total chlorophyll content in *BG757* line was 10% higher than in *A. thaliana* expressing PpDHN under salinity and drought stress [48], and greater than HbDHN1 and HbDHN2-expressing lines [18]. Heavy metals inhibit chlorophyll biosynthesis by disrupting key enzymes such as δ-aminolevulinic acid dehydratase and protochlorophyllide reductase [49]. The preservation of photosynthetic pigments in *BG757* line suggests that the bacterial dehydrin protects chloroplast structures and biosynthetic enzymes from heavy metal-induced damage, likely through ROS detoxification and protein stabilization mechanisms. This protection maintains photosynthetic capacity and energy production, supporting continued growth under stress.

BG757-expressing plants accumulated significantly higher proline levels under Cd and As stress (up to 23% higher than *PpDHN*-expressing lines under salinity and drought stress) [48]. Proline functions as an osmoprotectant, ROS scavenger, and molecular chaperone, stabilizing proteins and membranes under stress [50]. The coordinated upregulation of proline biosynthesis and dehydrin expression suggests synergistic stress protection mechanisms. Additionally, transgenic lines exhibited elevated total phenolic and flavonoid contents, which are critical components of the non-enzymatic antioxidant system [42]. These compounds scavenge ROS and reduce oxidative damage, complementing the protective functions of dehydrins [51]. The increased DPPH radical scavenging activity in *BG757* line confirms enhanced antioxidant capacity, which is essential for mitigating heavy metal-induced oxidative stress. Furthermore, transgenic plants maintained higher total protein content under stress, consistent with the chaperone function of dehydrins. Dehydrins prevent protein aggregation and degradation by interacting with partially denatured proteins through their amphipathic α-helices, forming molecular shields that stabilize protein structure [40,52]. This protective mechanism is particularly important under heavy metal stress, where ROS-induced protein oxidation and protease activation lead to extensive protein degradation [53]. In addition to ROS and antioxidant enzymes like SOD, other genes such as genes encoding PHYTOCHELATIN SYNTHASES (PCs), HEAVY METAL ATPase (HMA), CATION DIFFUSION FACILITATOR GENE FAMILY (CDF), Cd RESISTANCE GENE family, ATP-BINDING CASSETTE TRANSPORTER GENE family (ABC), METAL TOLERANCE PROTEINS (MTP1), and ethylene producing 1-AMINOCYCLOPROPANE-1-CARBOXYLIC ACID SYNTHASE (ACS) and 1-AMINOCYCLOPROPANE-1-CARBOXYLIC ACID OXIDASE (ACO) have also been implicated as important players in heacy metal tolerance [54]. MTP1 proteins and NATURAL RESISTANCE-ASSOCIATED MACROPHAGE PROTEINS (NRAMP) were also pointed out as important players for heavy metal tolerance in the hyperaccumulator *A. halleri*, as their genes are multiplicated and their expression is highly induced by heavy metal stress [55]. The higher performance of bacterial dehydrin BG757 compared to plant-derived dehydrins (HbDHN1, HbDHN2, PpDHN) in multiple physiological parameters (root length, chlorophyll content, proline accumulation) highlights the potential of cross-kingdom gene transfer for crop improvement. While the exact molecular basis for this enhanced performance requires further investigation, several factors may contribute: (1) structural differences in amphipathic helices and K-segments that enhance membrane binding and protein stabilization; (2) differential interaction with plant stress signaling pathways; and (3) unique sequence features that optimize chaperone activity in plant cellular environments. Recent studies suggest that dehydrin efficacy depends on the number and arrangement of conserved motifs (K-, Y-, and S-segments), which determine subcellular localization, client protein specificity, and stress-responsive expression patterns [45,46]. Future structural and functional analyses of BG757 will elucidate the molecular determinants of its higher protective capacity.

While this study demonstrates the efficacy of bacterial dehydrin BG757-1 in conferring heavy metal tolerance, several limitations warrant consideration. First, the molecular mechanisms underlying BG757’s better performance compared to plant dehydrins remain incompletely characterized. Detailed biochemical and structural studies are needed to identify specific protein–protein and protein–membrane interactions. Moreover, the extent of metal chelation by BG757 and its contribution to stress tolerance remain unclear, as some dehydrins confer protection without direct metal binding [40]. Additionally, the quantification of Cd and As accumulation in plant tissues was not performed in this study, which limits our understanding of metal uptake and distribution under stress conditions. Third, while our results demonstrate improved physiological parameters of in vitro grown plants, the downstream effects on crop yield, seed quality, and long-term stress adaptation require field-based validation. The prolonged in vitro growth is often suboptimal for plant growth, which can partly explain the slow root growth, may induce nutrient stress [56], and is different from field experiments with crop plants grown on soil. Alternative explanations for the observed stress tolerance include indirect effects of *BG757* expression on plant hormone signaling, altered expression of endogenous stress genes, or pleiotropic effects on plant development. Future studies employing transcriptomics, proteomics, and metabolomics approaches will provide comprehensive insights into *BG757*-mediated stress responses and identify additional targets for genetic improvement.

## 5. Conclusions and Future Recommendations

Meeting global food demand requires enhancing crop productivity under increasingly challenging environmental conditions. Heavy metal contamination, particularly from cadmium (Cd) and arsenic (As), significantly limits agricultural yields worldwide. This study demonstrates that bacterial dehydrin BG757 confers superior protection against Cd and As stress in transgenic *A. thaliana* compared to plant-derived late embryogenesis abundant (LEA) proteins. Transgenic lines exhibited enhanced growth parameters (root and shoot length, fresh and dry weight, leaf relative water content), elevated photosynthetic pigments, increased secondary metabolite production, higher total protein content, and improved antioxidant capacity (DPPH radical scavenging) under heavy metal stress. These findings establish bacterial dehydrins as a novel and potent resource for engineering abiotic stress tolerance in crops, offering a promising biotechnological approach to address food security challenges in contaminated agricultural systems.

## Data Availability

The original contributions presented in this study are included in the article. Further inquiries can be directed to the corresponding authors.
